# Treatment outcomes after uncomplicated and complicated crown fractures in permanent teeth

**DOI:** 10.1007/s00784-020-03344-y

**Published:** 2020-07-23

**Authors:** Ricarda Bissinger, Daniel David Müller, Marcel Reymus, Yegane Khazaei, Reinhard Hickel, Katharina Bücher, Jan Kühnisch

**Affiliations:** Department of Conservative Dentistry and Periodontology, University Hospital, Ludwig-Maximilians-University of Munich, Munich, Germany

**Keywords:** Complicated crown fracture, Dental trauma, Prognosis, Survival analysis, Treatment, Uncomplicated crown fracture

## Abstract

**Objectives:**

The objectives of this retrospective clinical study were to describe characteristics of crown fractures in permanent teeth and to investigate the survival of pulp vitality and restorations in uncomplicated and complicated crown fractures.

**Materials and methods:**

This retrospective study collected information from patients suffering from dental trauma who were treated between January 2004 and June 2017. The study population consisted of 434 patients (253 males/181 females; mean age 20.7 years) with 489 uncomplicated and 127 complicated crown fractures. The Kaplan-Meier survival curves and Cox proportional hazard regression analyses were performed to explore the data statistically.

**Results:**

The mean observation time was 522 days. Uncomplicated crown fractures without luxation showed a higher success rate of 82.3% (345/419) than complicated crown fractures without luxation (72.3%, 73/101). An additional luxation in uncomplicated crown fractures resulted in significantly reduced success rates in terms of survival of the pulp and restoration. Direct restorations survived significantly better independent of the fracture mode than did adhesively reattached crown fragments. No superiority of mineral trioxide aggregate or calcium hydroxide as pulp capping agent in complicated crown fractures was documented. Approximately 85.5% of all complications occurred within 2 years after the accident.

**Conclusion:**

The treatment of crown fractures resulted mostly in successful outcomes and only a moderate number of complications were observed.

**Clinical relevance:**

Primary dental management of crown fractures should follow recently published clinical guidelines, and close monitoring over at least 2 years seems to be justified.

## Introduction

Dental trauma is a frequent incident in permanent dentition and can occur in all stages of life, with increased numbers documented in the first and second decades of life [1, 2]. When considering the spectrum of injuries, crown fractures with or without pulp exposure are the most frequently recorded types of dental trauma [3, 4]. Numerous case reports and descriptive analyses have been published over the past few decades, which have mostly illustrated the clinical characteristics [5–8]. Unfortunately, few current longitudinal investigations [8–10] have included a large sample size or considered additional diagnoses, e.g. luxation or pulp exposure, and the corresponding treatments. Surprisingly, limited studies have been published on the clinical outcomes of bioactive cements, e.g. mineral trioxide aggregate (MTA), for pulp capping in teeth with complicated crown fractures [11], despite this material being widely used in daily dental practice. Therefore, information about its clinical performance is needed. Another unexpected finding was that only scarce longevity data are available for direct composite restoration in fractured anterior teeth [12]. Considering the previously mentioned gaps in knowledge, the objectives of this retrospective clinical study were to describe characteristics of crown fractures in permanent teeth and to provide longevity information about the survival of teeth, pulp, and restorations in relation to the fracture and luxation patterns. The null hypothesis formulated that the survival of pulp vitality and restoration would be equally distributed in relation to the type of injury.

## Materials and methods

This retrospective study was conducted in accordance with the Declaration of Helsinki. Ethical approval was obtained from the Human Ethics Committee of the Medical Faculty of the Ludwig-Maximilians-University, Munich (Project no. 670-16).

### Study population

This retrospective study collected information from patients suffering from dental trauma who were treated at the Department of Conservative Dentistry and Periodontology at Ludwig-Maximillians-University in Munich, Germany, between January 2004 and June 2017. Patients with a diagnosis of dental trauma in the primary or permanent dentition were included. Additional inclusion criteria were complete dental records on patient characteristics (e.g. age, gender) and detailed information about the traumatized tooth and corresponding treatment. Patients who received conservative treatment due to caries, periodontitis, developmental defects or aplasia were excluded. Finally, 898 patients (529 males/369 females; mean age of 16.9 years; range 0–86 years) with 1756 traumatized teeth (1344 permanent teeth/412 primary teeth; mean of 2.0 injured teeth per accident) were eligible for inclusion.

### Diagnostic and treatment principles at the Department of Conservative Dentistry and Periodontology

Key issues of the diagnostic and treatment protocols are described below. Importantly, any treatment decision was based on individual and standardized clinical and radiographic examinations. These examinations included sensitivity testing (method of first choice: refrigerant spray; method of second choice: electric pulp tester), an evaluation of the susceptibility to percussion, an assessment of tooth mobility and a compulsory apical radiograph for the detection of possible root fractures and for clarification of the status of root development. Furthermore, the dental hard tissue, endodontium, periodontium, alveolar bone and gingiva [13] were consistently assessed during the initial and recall visits. Diagnoses were made upon clinical and radiographic evaluations using the well-accepted classification system by Andreasen and Andreasen [14]. When crown fractures occurred, cases with no direct pulp exposure (uncomplicated crown fracture) and cases with pulp exposure (complicated crown fracture) were distinguished.

While uncomplicated crown fractures received mostly no indirect pulp treatment, the pulp tissue needed to be urgently preserved when the pulp was exposed. Here, bioactive cements, e.g. mineral trioxide aggregate (ProRoot MTA, Dentsply Sirona, York, PA, USA; Medcem MTA, Medcem GmbH, Wien, Austria), or an aqueous calcium hydroxide suspension was used for direct pulp capping. In general, for managing (un)complicated crown fractures, the clinicians followed the latest clinical guidelines, which were slightly modified during the study period [15–18]. Furthermore, in several cases, a root canal treatment was performed by the dentist who assumed the initial dental trauma care. Crown fractures with exposed pulp and fractures that were located near a pulp were immediately—at least on the same day the patient presented—treated with direct pulp capping or measurements for pulp protection (indirect pulp capping), respectively.

The management of dental defects in the hard tissue requires a deliberate restorative strategy. Two possible treatments were employed. First, if the fractured tooth fragment was saved, then the adhesive reattachment of the fragment [19] was the treatment procedure of choice. Second, if the fragment was lost, a direct composite restoration was applied. Again, the restorative management of (un)complicated crown fractures followed the latest clinical guidelines [15–18].

All diagnostic and therapeutic information were consistently recorded during the patient management on a separate case report form. After emergency treatment, each patient was offered a recall/monitoring visit at the appropriate interval according to the diagnosis at the Department of Conservative Dentistry and Periodontology.

### Standardized review of patient records

Two dental professionals (RB and DM) identified patients with any dental trauma based on the paper-based and electronic dental documentation records. Patients were considered if they presented in the Department of Conservative Dentistry and Periodontology during the interval from January 2004 to June 2017. A case report form was developed in which all relevant information regarding the dental trauma was entered to enable a structured data acquisition process: (1) patient characteristics (gender, age); (2) details of the dental trauma and its initial management (date, time, cause, circumstances and location of the accident, diagnoses, details of initial dental care); and (3) outcome variables (results from the monitoring visit, including complications, e.g. pulp vitality and survival of the restoration). Dental and/or medical reports or photographs were used as additional sources of information to obtain and/or verify details related to the dental trauma. All information was electronically acquired using data entry and documentation software (EpiData Software, version: V4.0.2.101, EpiData Association, EpiData - Comprehensive Data Management and Basic Statistical Analysis System, Odense, Denmark). If all the required information was not available or was misleading, the practitioner(s) was/were questioned to complete the missing information. In cases of diverging information, the study group (RB, DM and JK) reassessed the available data and discussed their points until a consensus was reached. In the case of undocumented details, the variable was missing.

### Statistical analysis

The whole dataset was initially transferred to an MS Excel sheet (Microsoft Office 365 Excel, version 1804, Redmond, WA, USA) via a csv file for further analysis. The descriptive analysis of the data was undertaken with Microsoft Excel and SPSS Statistics for Windows, Version 21.0.1 (SPSS Inc., an IBM Company, Armonk, NY, USA). Explorative statistical analysis was performed using R software (version 3.6.0, R Development Core Team, Vienna, Austria). The significance level was set at *α* = 0.05 with a 95% confidence interval. Survival curves were generated by Kaplan-Meier estimators [20]. For the survival analysis of the pulp vitality and the restorations, injuries were grouped into (un)complicated crown fractures with and without luxation. An assessment of the proportion of pulps and restorations that did not survive in a certain period after trauma and its treatment were linked to clinical situations indicating failure. The loss of pulp vitality was achieved when a tooth was classified as non-vital—with or without the presence of an apical inflammation—and was trepanned or received a root canal treatment. Restorations were recorded as failed if they were replaced because of loss or insufficiency, according to the FDI recommendations for the evaluation of direct restoration [21, 22]. In cases of reattached tooth fragments, failures were documented when the fragment was lost and was adhesively reattached. Differences in the survival rate were assessed by applying the log-rank test. Furthermore, a Cox proportional hazard regression analysis was performed to investigate the influence of the variables of interest on pulp and restoration survival.

## Results

Of the whole study population, 434 patients (253 males/181 females; mean age of 20.7 years; range 6–86 years) with a total of 616 teeth were affected by a(n) (un)complicated crown fracture (616/1344 teeth; 45.8% of all traumatized teeth in the permanent dentition). Of this population, 178 patients (41.0%) received emergency dental care at the Department of Conservative Dentistry and Periodontology, 222 patients (51.2%) presented to the Department of Conservative Dentistry and Periodontology for further treatment or monitoring after treatment by an external (dental) healthcare provider (mostly the Department of Oral and Maxillofacial Surgery), 4 patients (0.9%) requested a second opinion and the information was missing for 30 patients (6.9%). In detail, 489 (419 without luxation/70 with luxation) and 127 permanent teeth (101 without luxation/26 with luxation) were identified with an uncomplicated and complicated crown fracture, respectively. The upper central incisors were the most frequently fractured teeth in the permanent dentition (*N* = 439, 71.3%), followed by the upper lateral incisors (*N* = 109, 17.7%). Posterior teeth (*N* = 33, 5.4%), incisors in the mandible (*N* = 24, 3.9%) and canines (*N* = 11, 1.8%) were less frequently traumatized. An observable accumulation of fractured teeth was registered in patients aged between 6 and 16 years (*N* = 256; 41.6%). The mean observation time for all permanent teeth with crown fractures was 522.3 days (0–4837 days, 896.0 days standard deviation).

In cases of uncomplicated crown fractures, the tooth survival rate was 100%. In the group of complicated crown fractures, 5.5% (*N* = 7) of teeth were extracted.

The group of uncomplicated crown fractures without luxation (Table 1) comprised the largest proportion in the present sample, followed by complicated crown fractures without luxation (Table 2). Both entities showed high clinical success rates in terms of survival of pulp vitality and restoration. No loss of pulp vitality or restoration was observed in 82.3% (*N* = 345/419) and 72.3% (*N* = 73/101) of the teeth, respectively (Tables 1 and 2). In cases of additional luxation, the success rate decreased significantly (Table 3). When considering the loss of vitality, 27.1% of the pulps did not survive in the group of uncomplicated crown fractures with an additional luxation (Table 1); a similar order of magnitude (26.9%) was documented for complicated crown fractures (Table 2). The proportions of non-vital pulps amounted to 6.2% and 13.9% in the subjects with uncomplicated and complicated crown fractures without luxation, respectively. An additional luxation was found to be a significant disadvantageous factor for pulp survival in the Cox proportional hazard regression analysis in uncomplicated crown fractures. Interestingly, restorations on uncomplicated fractured teeth with an additional luxation performed better than those on non-dislocated teeth (Table 3).

**Table 1:** Overview of success rates and complication rates of pulp and restorative treatments for uncomplicated crown fractures with and without accompanying luxation injuries

Diagnosis	Pulp treatment	Restorative treatment	Success		Complications					
					Loss of vitality		Loss of restoration		Total	
			*N*	%	*N*	%	*N*	%	N*	%
Uncomplicated crown fracture without luxation (*N* = 419)	Indirect pulp capping	Reattachment (*N* = 6)	4	66.7	2	33.3	–	–	2	33.3
		Direct restoration (*N* = 20)	13	65.0	3	15.0	5	29.4	7	35.0
	No additional pulp protection	Reattachment (*N* = 67)	48	71.6	1	1.5	18	26.9	19	28.4
		Direct restoration (*N* = 298)	253	83.6	19	6.4	28	9.4	45	15.1
		No restoration (*N* = 28)	27	96.4	1	3.6	–	–	1	3.6
		**∑**	345	82.3	26	6.2	51	12.2	74	17.7
Uncomplicated crown fracture with luxation (*N* = 70)	Indirect pulp capping	Reattachment (*N* = 3)	3	100.0	–	–	–	–	–	–
		Direct restoration (*N* = 4)	2	50.0	1	25.0	1	25.0	2	50.0
	No additional pulp protection	Reattachment (*N* = 8)	6	75.0	1	12.5	1	12.5	2	25.0
		Direct restoration (*N* = 46)	29	63.0	16	34.8	1	2.2	17	37.0
		No restoration (*N* = 9)	8	88.9	1	11.1	–	–	1	11.1
		**∑**	48	68.6	19	27.1	3	4.3	22	31.4

*One tooth may have multiple complications

**Table 2:** Overview of success rates and complication rates of pulp and restorative treatments for complicated crown fractures with and without accompanying luxation injuries

Diagnosis	Pulp treatment		Restorative treatment	Success		Complications					
						Loss of vitality		Loss of restoration		Total	
				*N*	%	*N*	%	*N*	%	N*	%
Complicated crown fracture without luxation (*N* = 101)	Direct pulp capping	MTA	Reattachment (*N* = 13)	9	69.2	–	–	4	30.8	4	30.8
			Direct restoration (*N* = 9)	6	66.7	3	33.3	–	–	3	33.3
		Ca(OH)_2_	Reattachment (*N* = 9)	4	44.4	4	44.4	3	33.3	5	55.6
			Direct restoration (*N* = 14)	9	64.3	3	21.4	3	21.4	5	35.7
	Pulpotomy	MTA	Reattachment (*N* = 5)	3	60.0	1	20.0	2	40.0	2	40.0
			Direct restoration (*N* = 2)	1	50.0	–	–	1	50.0	1	50.0
		Ca(OH)_2_	Reattachment (*N* = 4)	2	50.0	2	50.0	1	25.0	2	50.0
			Direct restoration (*N* = 0)	–	–	–	–	–	–	–	–
	Root canal treatment		Reattachment (*N* = 7)	5	71.4	–	–	2	28.6	2	28.6
			Direct restoration (*N* = 17)	14	82.4	–	–	3	17.6	3	17.6
	Not specified		Reattachment (*N* = 3)	2	66.7	1	33.3	–	–	1	33.3
			Direct restoration (*N* = 11)	11	100.0	–	–	–	–	–	–
	Extraction		(*N* = 7)	7	100.0	–	–	–	–	–	–
			∑	73	72.3	14	13.9	19	18.8	28	27.7
Complicated crown fracture with luxation (*N* = 26)	Direct pulp capping	MTA	Reattachment (*N* = 5)	1	20.0	1	20.0	4	80.0	4	80.0
			Direct restoration (*N* = 4)	1	25.0	3	75.0	1	25.0	3	75.0
		Ca(OH)_2_	Reattachment (*N* = 0)	–	–	–	–	–	–	–	–
			Direct restoration (*N* = 2)	2	100.0	–	–	–	–	–	–
	Pulpotomy	MTA	Reattachment (*N* = 3)	3	100.0	–	–	–	–	–	–
			Direct restoration (*N* = 0)	–	–	–	–	–	–	–	–
		Ca(OH)_2_	Reattachment (*N* = 2)	1	50.0	–	–	1	50.0	1	50.0
			Direct restoration (*N* = 0)	–	–	–	–	–	–	–	–
	Root canal treatment		Reattachment (*N* = 0)	–	–	–	–	–	–	–	–
			Direct restoration (*N* = 5)	5	100.0	–	–	–	–	–	–
	Not specified		Reattachment (*N* = 0)	–	–	–	–	–	–	–	–
			Direct restoration (*N* = 5)	2	40.0	3	60.0	–	–	3	60.0
	Extraction		(*N* = 0)	–	–	–	–	–	–	–	–
			∑	15	57.7	7	26.9	6	23.1	11	42.3

*One tooth may have multiple complications

**Table 3:** Results from the logistic regression analysis which analyses potential associations between uncomplicated and complicated crown fractures and injury or treatment relevant covariables

	Loss of pulp vitality		Loss of restoration	
	Hazard ratio (95% CI)	*p* value	Hazard ratio (95% CI)	*p* value
Uncomplicated crown fractures				
Age	0.99 (0.98–1.02)	0.729	1.00 (0.99–1.02)	0.558
Gender				
Male	1.00		1.00	
Female	1.50 (0.81-2.76)	0.209	0.95 (0.54-1.69)	0.864
Periodontal diagnosis				
No luxation	1.00		1.00	
Luxation	*4.61* (*2.47*–*8.61*)	< *0.001*	*0.30* (*0.10*–*0.98*)	*0.046*
Pulp protection				
None	1.00		1.00	
Indirect pulp capping	1.79 (0.74-4.35)	0.196	1.36 (0.57-3.22)	0.483
Restorative treatment				
Reattachment	1.00		1.00	
Direct restoration	2.29 (0.80-6.51)	0.121	*0.35* (*0.19*–*0.62*)	*0.000*
Complicated crown fractures				
Age	*1.10* (*1.03*–*1.17*)	*0.007*	1.01 (0.96-1.07)	0.655
Gender				
Male	1.00		1.00	
Female	2.46 (0.85-7.08)	0.096	1.12 (0.47-2.64)	0.804
Periodontal diagnosis				
No luxation	1.00		1.00	
Luxation	0.97 (0.27-3.52)	0.964	1.10 (0.40-2.97)	0.859
Pulp treatment				
Root canal treatment	–	–	1.00	
Direct pulp capping (Ca(OH)_2_)	1.00		0.75 (0.22-2.61)	0.654
Direct pulp capping (MTA)	1.72 (0.47-6.25)	0.413	0.99 (0.28-3.53)	0.992
Pulpotomy (Ca(OH)_2_)	2.43 (0.35-16.94)	0.368	0.50 (0.08-3.18)	0.463
Pulpotomy (MTA)	0.48 (0.05-4.41)	0.517	0.55 (0.11-2.72)	0.459
Restorative treatment				
Reattachment	1.00		1.00	
Direct restoration	1.42 (0.48-4.21)	0.526	*0.37* (*0.14–0.93*)	*0.034*

Italic numbers indicate a significant association

A separate analysis investigated the outcomes of the use of MTA and calcium hydroxide as pulp capping agents in patients with complicated crown fractures. Regarding the percent loss of vitality, more vital pulps (80.5%, *N* = 33/41) were observed in the MTA group than in the calcium hydroxide group (71.0%, *N* = 22/31). When considering the results from the Cox proportional hazard regression analysis, a non-significant influence of the clinical pulp treatment was documented (Table 3).

A higher proportion of lost restorations was observed in the group with complicated crown fractures than in the group with uncomplicated crown fractures (Tables 1 and 2). Furthermore, direct restorations performed significantly better than reattached tooth fragments independent of the mode of fracture (Table 3).

**Fig. 1:**
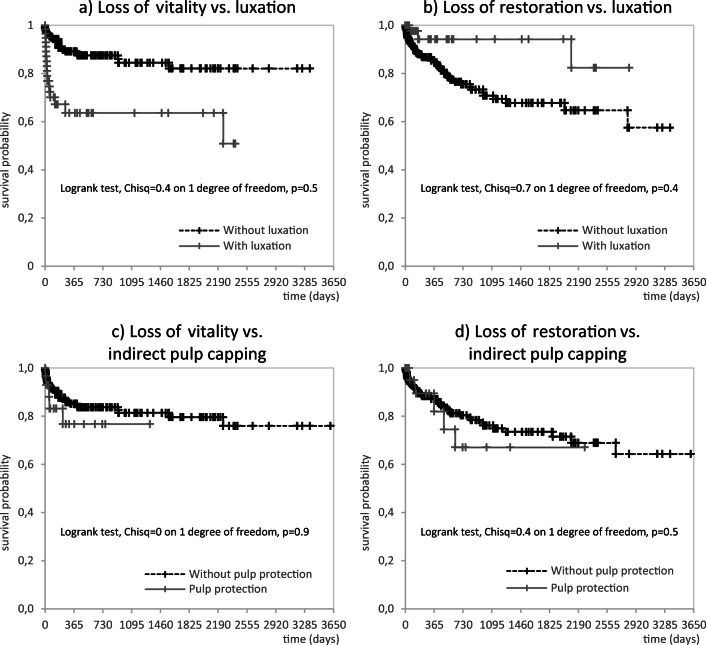
The Kaplan-Meier curves for uncomplicated crown fractures illustrate the pulpal survival probability and longevity of direct restorations in relation to additional luxation injuries (**a**, **b**) and the use of indirect pulp capping (**c**, **d**)

**Fig. 2:**
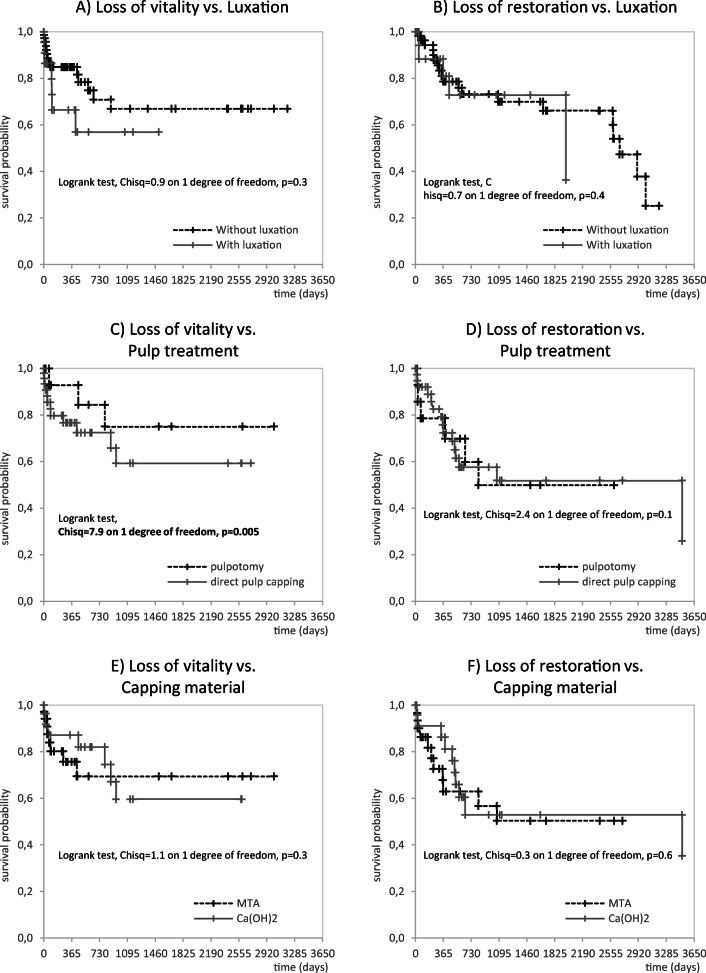
The Kaplan-Meier curves for complicated crown fractures illustrate the pulpal survival probability and longevity of direct restorations in relation to additional luxation injuries (**a**, **b**), the indicated pulp treatment (**c**, **d**) and used pulp capping material (**e**, **f**)

The mean time interval of pulp failure was 338.8 days (standard deviation 735.9 days) and 280.9 days (sd 371.1 days) in the groups with uncomplicated and complicated crown fractures, respectively. In cases of a restoration failure, the corresponding mean intervals were 469.2 days (sd 723.9 days) for uncomplicated crown fractures and 310.4 days (sd 357.3 days) for complicated crown fractures. Overall, 85.5% of all complications—loss of pulp vitality and/or restoration—occurred within the first 2 years after the accident (Table 4). The Kaplan-Meier curves which estimate survival from lifetime data are shown in Figs. 1 and 2.

**Table 4:** Overview of the intervals before complications occurred with respect to the loss of vitality or loss of restoration

Diagnosis	Time (years)	Loss of vitality			Loss of restoration		
		*N*	%	Cum. %	*N*	%	Cum. %
Uncomplicated crown fracture without luxation (*N* = 419)	< 0.5	14	53.8	53.8	26	51.0	51.0
	0.5–1.0	5	19.2	73.1	5	9.8	60.8
	1.1–1.5	2	7.7	80.8	8	15.7	76.5
	1.6–2.0	1	3.8	84.6	3	5.9	82.4
	> 2.0	4	15.4	100.0	9	17.6	100.0
	∑	26	100.0	100.0	51	100.0	100.0
Uncomplicated crown fracture with luxation (*N* = 70)	< 0.5	16	84.2	84.2	2	66.7	66.7
	0.5–1.0	1	5.3	89.5	–	–	–
	1.1–1.5	–	–	–	–	–	–
	1.6–2.0	–	–	–	–	–	–
	> 2.0	2	10.5	100.0	1	33.3	100.0
	∑	19	100.0	100.0	3	100.0	100.0
Complicated crown fracture without luxation (*N* = 101)	< 0.5	9	64.3	64.3	9	47.4	47.4
	0.5–1.0	–	–	–	5	26.3	73.7
	1.1–1.5	2	14.3	78.6	2	10.5	84.2
	1.6–2.0	–	–	–	2	10.5	94.7
	> 2.0	3	21.4	100.0	1	5.3	100.0
	∑	14	100.0	100.0	19	100.0	100.0
Complicated crown fracture with luxation (*N* = 26)	< 0.5	5	71.4	71.4	3	50.0	50.0
	0.5–1.0	1	14.3	85.7	1	16.7	66.7
	1.1–1.5	1	14.3	100.0	1	16.7	83.3
	1.6–2.0	–	–	–	–	–	–
	> 2.0	–	–	–	1	16.7	100.0
	∑	7	100.0	100.0	6	100.0	100.0

## Discussion

This retrospective clinical study provides detailed data about the outcomes and prognosis of dental management for fractured permanent teeth and represents an update of an earlier report [9]. Uncomplicated and complicated crown fractures, representing 45.8% (*N* = 616/1344 teeth) of all traumatic events in the present sample, comprised the largest proportion of dental injuries of the permanent dentition, consistent with the literature [23]. The key information from this study is that crown fractures and their clinical management were generally linked to acceptably high success rates. Nevertheless, in some situations, a loss of pulp vitality and/or restoration will occur. Therefore, the initially formulated hypothesis needs to be rejected.

A common complication of endodontic treatment is a loss of pulp vitality, which was more frequently detected in cases with additional luxation (Tables 1 and 2); this association was found to be significant in uncomplicated crown fractures (Table 3). This finding is easily explained by two factors. First, it might be caused by the traumatization of the coronal dentin-pulp system, resulting in a perforation of dentin tubules or in the direct opening of the pulp; in both situations, the odontoblast cells might be negatively affected. Second, the pulp vitality might be damaged due to the temporary or definitive interruption of the blood supply at the foramen apicale, which is typically linked to luxation injuries. Both scenarios might be associated with a potential loss of pulp vitality after the dental trauma. When viewing our data in this context, notable trends were observed (Tables 1 and 2). The highest pulp survival rate was documented in teeth with uncomplicated fractures without luxation (93.8%, Table 1), followed by complicated fractures without an additional luxation (86.1%, Table 2). These numbers appear to be identical to or above-average compared to previously published data, which indicated loss of vitality in up to 24% of all cases [3, 11, 24]. The proportion of endodontic complications increased in our sample when a luxation was additionally diagnosed; approximately one of four teeth lost its vitality within the observation period (Tables 1 and 2), which is a biologically plausible finding and illustrates the clinical relevance of an additional luxation injury. Interestingly, few and heterogeneous data have been published from comparable studies to date and have documented more favourable [8], equal [8] or unfavourable results [3, 5–7, 10].

When considering the clinical success of pulp therapy in relation to the capping material, MTA tended to provide better but non-significant clinical results than calcium hydroxide in the case of indicated pulpotomies. Interestingly, an inverse trend was documented in the case of direct pulp capping. Nevertheless, no superiority of a pulp-maintaining procedure can be derived from the Cox proportional hazard regression analysis of the current data (Table 3). When considering the clinical advantages of bioactive cements [25–28], there might be a clinical preference from the current point of view to use such products. Nevertheless, the use of calcium hydroxide results in similar clinical success rates. In addition, the findings from our data should not be overstated due to the observational character of this investigation and the low case numbers in some categories and must be verified in future clinical trials.

Another relevant question for clinicians regards the possible time point when an endodontic failure may occur. Here, 64.3 to 89.5% of all documented endodontic events—loss of pulp vitality—occurred within the first year after dental trauma (Table 4). At least 78.6% of all endodontic events were diagnosed 2 years after the accident (Table 4), which is further illustrated in the Kaplan-Meier curves (Figs. 1 and 2). Based on this finding, a quarterly follow-up of crown fractures over 2 years seems to be indicated. Recall intervals may be prolonged 2 years after the trauma, as only few cases of complications appeared thereafter.

Another frequently diagnosed complication was the loss of restoration. Interestingly, the proportions were higher in the group with complicated crown fractures than in the group with uncomplicated crown fractures (Tables 1 and 2; Figs. 1 and 2). These numbers were explained by the fact that complicated crown fractures are frequently linked to a more severe hard tissue defect, which requires more extensive restoration than uncomplicated crown fractures. In contrast, restorative treatment in teeth with uncomplicated crown fractures exhibited the highest survival probability (Tables 1 and 2). Another clinically relevant finding from this report is that direct restorations performed better than reattachment of tooth fragments. This finding needs to be carefully interpreted because the use of crown fragments should be considered the restorative method of choice due to its minimal invasiveness and natural aesthetics [29, 30]. In contrast to this finding, the use of direct composite restorations is linked to higher survival rates because of the probability of bevelling the restoration margins and enlarging adhesions in the enamel. With respect to these important clinical variables, the documented survival rates (Table 3) should not be misinterpreted to favour direct restorations in primary dental trauma care. Finally, it can be concluded from our data in comparison to existing information from the literature [31–33] that restorations placed in the present study were associated with high survival rates. When reporting the longevity of restorations, the clinician must also know the time intervals in which losses of restoration may occur. Within the first years after the primary restoration was placed, 60.8 to 73.7% of all failures were diagnosed. These numbers increased to 66.7 to 94.7% of failed restorations after 2 years (Table 4). Even in the case of a possible loss of restoration, the previously mentioned recommendation to monitor traumatized teeth closely over 2 years needs to be repeated.

A worst-case scenario, which never occurred during the observation period, was the indication for extraction of any permanent teeth. This finding indicates a primarily successful dental management of crown fractures, which was also documented in other studies [8, 23, 34]. Nevertheless, seven teeth with a documented complicated crown fracture were removed by the dentist who was responsible for emergency care. This decision was part of the initial treatment and was not the result of complications during the observation period after conservative therapy. Here, we presumed that these teeth experienced multiple damages, and the prognosis was therefore probably assessed as poor. All patients with extractions had been referred for further treatment by private or other external (dental) healthcare providers. When considering the proportion of patients who were not treated at the Department of Conservative Dentistry and Periodontology, this retrospective, practice-based analysis also included patients who were probably not always treated according to the latest treatment protocols; thus, heterogeneity in the treatment protocols used by dentists in dental practices and dentists at the Department of Conservative Dentistry and Periodontology might exist.

The strength of the present retrospective, longitudinal case-control study is the large sample size and detailed analysis of data, including the presence or absence of a luxation injury. Furthermore, all included patients were treated after the turn of the millennium, indicating that treatment decisions were made based on recently published clinical guidelines [16–18]. Based on the results from the survival analyses (Figs. 1 and 2), valuable information about the prognosis of (un)complicated crown fractures was provided.

Some limitations of this investigation are related to the study design. In general, a prospective study design would ensure more reliable data but is difficult to achieve since the participation of the patient must already be obtained in an emergency situation, which is questionable from an ethical perspective [20]. Therefore, retrospective analyses are frequently used in dental traumatology [3, 4, 23, 25, 35–37] but have several limitations. Most importantly, all information was only collected from patient records, radiographs, photographs, doctor’s letters or trauma documentation sheets. Thus, information was missing for some patients, which was not compensated from other sources of information. Furthermore, the present samples of patients with dental trauma might have been influenced by the different levels of clinical experience and skills of dental practitioners at the primary dental care facility. In this study, approximately 20 doctors treated patients in the Department of Conservative Dentistry and Periodontology at different frequencies. Therefore, divergent diagnostic and treatment decisions might have occurred, which may limit the informational value of the present report. In addition to the heterogeneity of practitioners, the changes in treatment policies [16–18] over the years must also be considered. When considering this study, patients receiving non-IADT guideline-compliant treatments may also be present in this pool of patients/data. Nearly 40% of the patients initially received treatment in the Department of Conservative Dentistry and Periodontology, and the remaining proportion was referred to the department after emergency treatment. Therefore, we were sometimes unable to collect detailed information about the IADT guideline-conforming initial treatments for the latter group.

A further limitation of practice-based, retrospective studies is that the study design did not include strict recall intervals after the initial treatment visit. While many patients adhered to the recommendations of dental team at the University Hospital, several other patients asked other professionals to perform the monitoring after the dental trauma and were, therefore, lost to follow-up in the present study. Consequently, the number of patients with long follow-up times decreased with an increasing observation time.

## Conclusions

Regarding the overall number of traumatic events in the permanent dentition, crown fractures are a frequent injury that must be appropriately addressed by the general dental practitioner. When considering that most of the contemporarily recommended treatment protocols by the IADT were applied to the included patients and the high survival rates, it can be concluded that these clinical procedures are linked with successful treatment outcomes in the majority of patients. In addition, the following conclusions can be drawn: (1) the loss of pulp vitality should be considered a potential complication, which might be more likely to occur in cases of an additional luxation injury; (2) no statistically significant superiority of MTA or calcium hydroxide as pulp capping material in cases of complicated crown fracture was detected; (3) restoration failure was more frequently detected in reattached crown fragments; and (4) based on the registered survival rates and the fact that most complications occurred within the first 2 years after dental trauma, close clinical monitoring intervals over the first 24 months after the initial trauma are justified.
